# Supplementary material for: Kinematic Behavior of an Untethered, Small-Scale Hydrogel-Based Soft Robot in Response to Magneto-Thermal Stimuli
Source: Biomimetics (Basel). 2023 Aug 19;8(4):379. doi: 10.3390/biomimetics8040379 (PMC10452115; doi:10.3390/biomimetics8040379)
Supplement: Supplementary file 1 [file biomimetics-08-00379-s001.zip › biomimetics-2514590-supplementary.pdf]

Supplementary materials

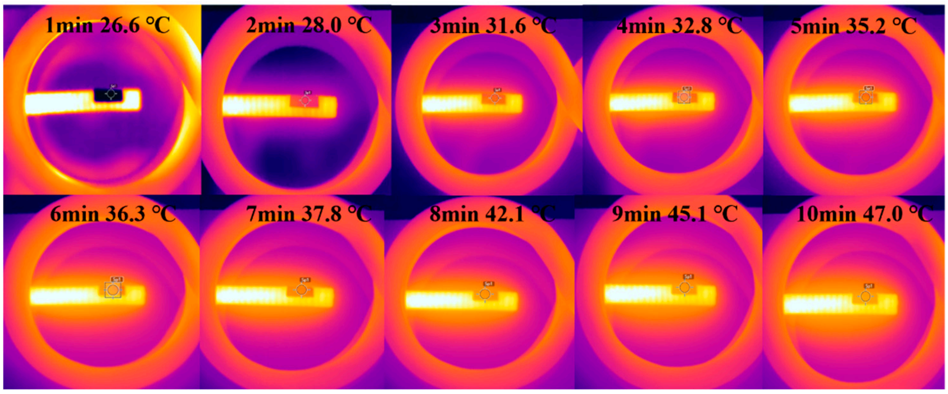

Figure S1. Infrared image of a gastropod-free soft robot walking on a gastropod microstructure.

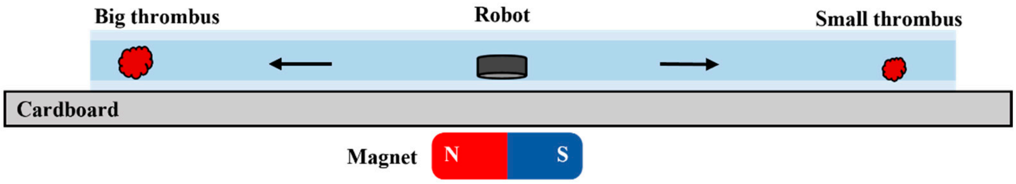

Figure S2. Schematic diagram of magnetic navigation motion platform.

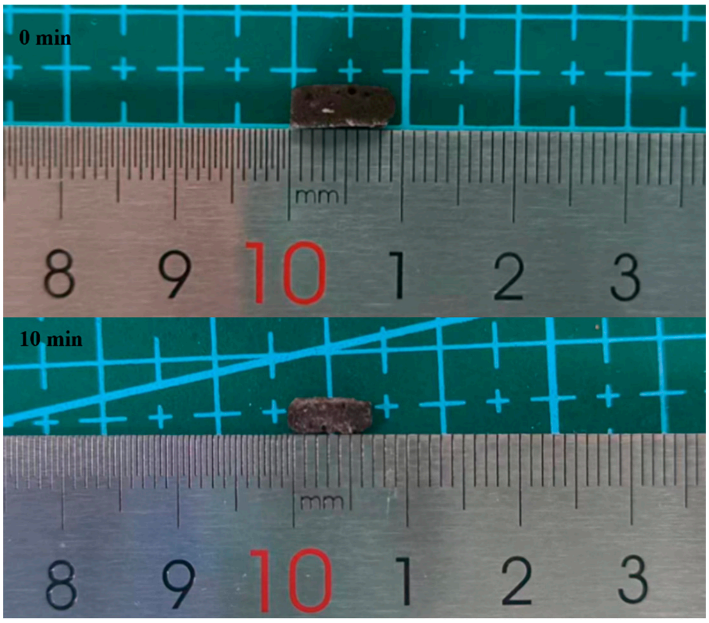

Figure S3. Size change of the robot under 28-A induced current.
